# Using Interoperability between Mobile Robot and KNX Technology for Occupancy Monitoring in Smart Home Care

**DOI:** 10.3390/s23218953

**Published:** 2023-11-03

**Authors:** Jan Vanus, Radim Hercik, Petr Bilik

**Affiliations:** Department of Cybernetics and Biomedical Engineering, VŠB-TU Ostrava, 70800 Ostrava, Czech Republic; radim.hercik@vsb.cz (R.H.); petr.bilik@vsb.cz (P.B.)

**Keywords:** Smart Home Care, mobile robot, occupancy, presence, localization, KNX technology

## Abstract

It is important for older and disabled people who live alone to be able to cope with the daily challenges of living at home. In order to support independent living, the Smart Home Care (SHC) concept offers the possibility of providing comfortable control of operational and technical functions using a mobile robot for operating and assisting activities to support independent living for elderly and disabled people. This article presents a unique proposal for the implementation of interoperability between a mobile robot and KNX technology in a home environment within SHC automation to determine the presence of people and occupancy of occupied spaces in SHC using measured operational and technical variables (to determine the quality of the indoor environment), such as temperature, relative humidity, light intensity, and CO_2_ concentration, and to locate occupancy in SHC spaces using magnetic contacts monitoring the opening/closing of windows and doors by indirectly monitoring occupancy without the use of cameras. In this article, a novel method using nonlinear autoregressive Neural Networks (NN) with exogenous inputs and nonlinear autoregressive is used to predict the CO_2_ concentration waveform to transmit the information from KNX technology to mobile robots for monitoring and determining the occupancy of people in SHC with better than 98% accuracy.

## 1. Introduction

Globally, the number of older people and the demand for assistive care is growing rapidly [1]. The number of older people with unmet care and support needs is increasing significantly due to the challenges faced by formal and informal care systems in different countries. The World Health Organization (WHO) suggests that the development of intelligent, physical, social, and age-friendly environments will improve the quality of life of older people. Current technological innovations and developments have the potential to address some of the challenges in the care and support of older people [2]. Assistive technologies have been an important topic in both research and manufacturing industries in recent decades, with a focus on improving social interaction and supporting healthcare, business, education, and activities of daily living [3]. There are different types of assistive technologies such as assistive autonomous robots, self-driving vehicles, smart health applications, wearable devices with Artificial Intelligence (AI) support, novel drug release mechanisms, wearable diagnostics, voice-activated devices, virtual, augmented, and mixed reality, or building automation technologies in smart buildings [2]. Considering the situation with the COVID-19 pandemic in the world [4], Social Companion Robots (SCRs) integrated with various sensing technologies such as vision, voice, or touch to interact with other smart devices in the home can enable the development of advanced artificial intelligence solutions towards Smart Home Care with assistive services to address the needs of the elderly and disabled [1]. The knowledge of the spatial distribution of physical quantities in Smart Home (SH) supports the development of new context-aware applications where new methodologies for multi-sensor data fusion and information processing are needed [5], with possible applications such as multi-point path planning for a home mobile robot [6]. The increasing isolation of elderly people in their own homes and in homes for the elderly has made the problem of caring for the elderly living alone an urgent priority. Experiments conducted using robots in the home support the conclusion that an assistive robot can correctly design activities to ensure a good quality of life for the elderly [7]. Within the Smart Cities platform, robots can perform simple tasks in hotels as hotel receptionists, museum guides, waiters in cafes and restaurants, home assistants, and others [8]. Robotics, artificial intelligence, and the Internet of Things support various processes in many scenarios of modern life, such as e-health [9] and psychological treatment [10]. Robotic doctors and nurses developed through IoT technology will provide healthcare to patients in the future [11]. Brunete, A. et al. present a new architecture that integrates Internet of Things (IoT) devices, service robots, and users into an intelligent assistive environment that supports disabled and bedridden patients [12,13,14].

In the presented study, a unique hardware and software solution is newly described to ensure mutual communication and interoperability between KNX technology (standard EN 50090, ISO/IEC 14543 [15] for building automation) and an autonomous mobile robot (AMR) [16,17,18,19,20,21,22] designed to serve and assist Smart Home Care residents in the framework of social assistance robotics for indirect monitoring of occupancy of SHC premises and indirect localization of people without the use of cameras. The proposed solution uses the KNX open data protocol to provide data connectivity with other technologies. The Smart Home Care technology concept is used to support independent living for the elderly and disabled in the home environment. Different technologies and software (SW) implementation can be used for the automation of intelligent buildings in Smart Home Care (Table 1).

The presented work describes a new unique solution, which is based on the use of AMR in SHC for the localization of people, monitoring the presence of people, and determining the occupancy of the monitored spaces.

The newly designed indirect method was used for follow-up actions (resulting from the set mode of operation of the AMR) from measured operational variables to determine the quality of the indoor environment (CO_2_) and variables within the monitoring of the opening of windows and doors (magnetic contacts) in the framework of building automation used KNX technology. In the framework of the newly proposed method, Nonlinear Autoregressive (NAR) and Nonlinear Autoregressive Exogenous (NARX) Neural Network (NN) with different network settings have been used for CO_2_ waveform prediction.

**Figure 1:**
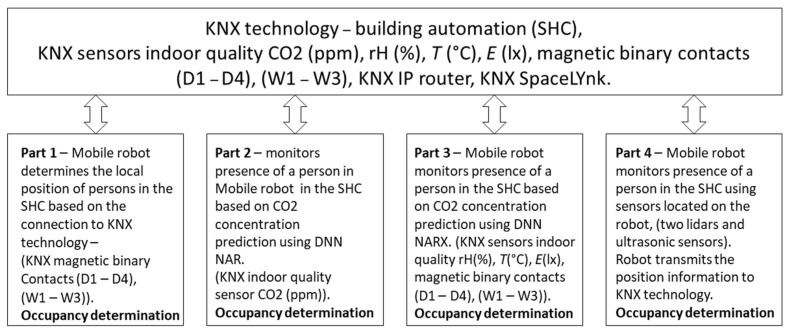
Block Diagram—Interoperability between KNX and AMR Technologies.

Research contribution—The research contribution contains four main parts as follows (Figure 1):

- Part 1. AMR locates the position of a person using an indirect method (without the use of cameras) based on the connection to KNX technology—opening/closing a window (W1, W2, W3) or a door (D1, D2, D3, D4).
- Part 2. AMR indirectly monitors (without a camera) the presence of a person in a room ahead of time based on the prediction of CO_2_ concentration using the neural network NAR with advance and subsequent transmission of information to AMR without a camera (indirect determination of occupancy of monitored spaces in SHC).
- Part 3. The AMR indirectly monitors (without camera) the presence of a person in a room ahead of time based on the prediction of the CO_2_ concentration waveform in advance using the measured KNX operational technical variables such as indoor temperature, indoor relative humidity, indoor light intensity, window opening/closing information (W1, W2, and W3), and door opening/closing information (D1, D2, D3, and D4) using the NARX neural network and subsequent transmission of the information to the AMR.
- Part 4. The presence of people is monitored in the SHC using sensors placed on the AMR, specifically two lidars and ultrasonic sensors. Monitoring is based on the principle of detecting obstacles in the map that are not normally there. By comparing the map background and the sensor data, a moving object can also be detected (here, an example of a blank map and a map where a person is detected can be provided).

The proposed architecture will be applied to an existing decentralized KNX technology in a smart home environment for the purpose of monitoring and monitoring the occupancy of individual SHC rooms. The paper is structured as follows: Section 2 introduces relevant studies and key technologies in smart home solutions using the SHC platform with KNX and AMR; Section 3 describes AMR; Section 4 introduces KNX technology designed for SHC automation (indoor environment quality monitoring, security functions of opening/closing windows, and doors); Experimental Section 5 is divided into Part 1 KNX-AMR (localizes the position of a person), Part 2 KNX-AMR (monitors room occupancy indirectly with time advance using CO_2_ concentration prediction using NAR neural network), Part 3 KNX-AMR (monitors room occupancy indirectly with time advance using CO_2_ concentration prediction using NARX neural network), Part 4(monitoring the presence of people in SHC using sensors placed on AMR description of experiments); Section 6 compares NAR and NARX NNs in an application to determine the presence of people in a monitored room; Section 7 concludes, evaluates, and outlines the next steps in solving the described problem.

## 2. Related Work

Assistive technology has been a significant topic in both the research and manufacturing industry for the past decades focusing on improving social interaction, supporting health care, business, education, and daily activities. There are different types of assistive technologies such as wearable devices, mobile applications, automated home appliances, and robots [1] (socially assistive robotics (SAR). The increasing isolation of the elderly both in their own homes and in care homes has made the problem of caring for elderly people who live alone an urgent priority [2]. However, for robotic assistive applications to be effective, they need to satisfy the particular needs of each user and be well-perceived [3]. A rising proportion of older people has more demand for services including hospitals, retirement homes, and assisted living facilities [4]. A summary of state-of-the-art mobile robot implementations in smart home care automation is presented in Table 1.

Different technologies and software (SW) resources can be used for the Smart Home Care building automation, such as KNX technology [33,34,35,36,37], FPGA technology [38], IoT platform [39], STM32F762 [40], VB-PSO [41], Feed-forward [42], LabVIEW [43], Raspberry PI [44], or PC STC89C52 with additional sensors [45].

None of the articles mentioned in the above research (Table 1) describes a comprehensive solution to provide intercommunication and interoperability between KNX technology designed for building automation and AMR designed to serve and assist SHC occupants, using it for indirect occupancy monitoring of SHC premises and indirect occupant localization without the use of cameras.

The presented study describes a new unique solution for the use of AMR in SHC for occupant localization, monitoring the presence of people, and determining the occupancy of the monitored spaces using an indirect method without the use of cameras for subsequent actions (resulting from the set mode of operation of the AMR) based on the measured operational variables for determining the quality of the indoor environment [46,47,48,49,50,51,52] and operational variables for the building security area—opening/closing windows and doors within the automation of the SHC using KNX technology with the use of CO_2_ prediction using the nonlinear autoregressive recurrent dynamic neural network (NN) with exogenous inputs (NARX), which is used for time-series modeling and nonlinear autoregressive (NAR) NN:

- The AMR determines the position of the person (information sent from the KNX technology);
- AMR monitors the presence of a person in the SHC in advance (information sent from KNX technology), NN (NARX, NAR);
- AMR provides monitoring of the presence of persons in the SHC using sensors placed on the robot, namely two lidars and ultrasonic sensors (information sent to KNX technology).

## 3. Autonomous Mobile Robot

**Figure 2:**
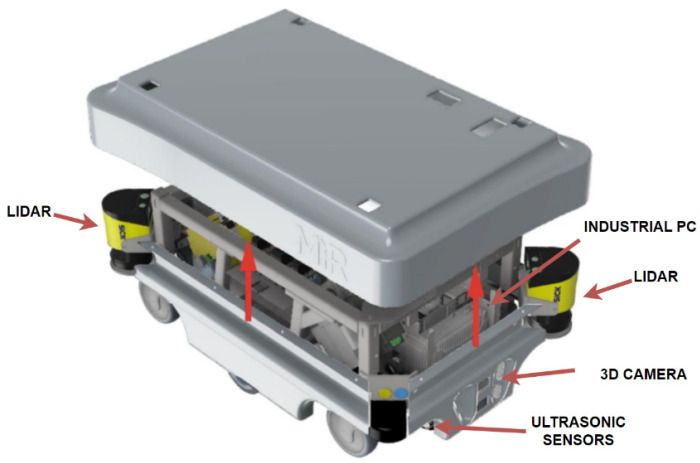
Mobile industrial robot MiR100 [53].

An autonomous mobile robot (AMR) is an industrial device containing a sensing part and an actuating part (motors). It also contains an industrial control computer with a Linux operating system, a 24 V Li-ion battery with a capacity of 0.9 kWh, and other sensorics safety and support systems as shown in Figure 2. The sensor system consists mainly of two lidars from Sick, placed diagonally, as well as ultrasonic sensors at the front and rear of the robot and proximity sensors monitoring the robot’s immediate surroundings.

**Figure 3:**
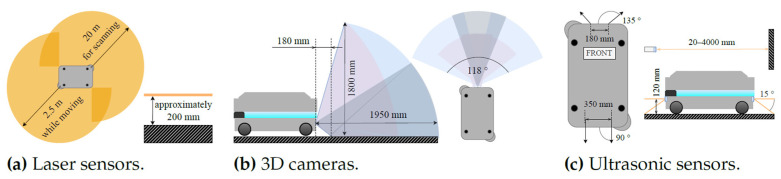
Obstacle detection system [53].

The AMR is capable of autonomous movement in a given space based on a virtual map of the environment, which it compares with the data collected by sensors (Figure 3).

### 3.1. Robot Specification

For the purpose of this thesis, the AMR developed by the Danish company MiR was selected. Specifically, it is the MiR100 model. It is the smallest robot in the portfolio with dimensions of 890 mm in length, 580 mm in width, and 352 mm in height and a total payload of 100 kg. Due to its compact size, the robot can be used in the home and in apartments. At the same time, its load capacity is sufficient for transporting people with reduced mobility or larger loads.

### 3.2. Navigation System of Robot

In order to find a route, the navigation system needs initialization data to plan the route. The user enters information about where the robot should arrive. However, in order for AMR to plan a route, it also needs to know the space it is moving in and its current location. A virtual map is used to navigate the space, which is stored in the robot’s memory and contains information about all walls and obstacles. This map must be created before the AMR can be used. Once the virtual map is created, the points on which the robot moves can be inserted. However, the map can be modified with zones, landmarks, and special planning rules.

Once all the data is obtained, route planning is initiated. The route is planned by the robot itself, which is in charge of the global planning system. It is an algorithm that generates the route to the desired point. However, it is important that the global planning system generates the path to the destination only once and only tracks fixed obstacles that are recorded on the virtual space map. This means that if a new obstacle appears in the robot’s environment that is not recorded on its virtual space map, the global planning system is unaware of this obstacle and plans the path despite this obstacle. The planned route is displayed on the map or on the dashboard using points.

**Figure 4:**
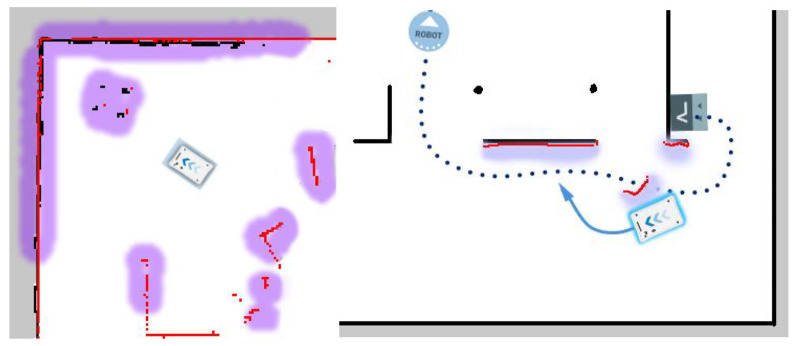
Example diagram to demonstrate the AMR automatic route planning in practice.

Figure 4 shows an example of robot navigation on a virtual map. The black contours represent fixed obstacles such as walls, whereas the red contours represent fixed obstacles detected by the robot. The purple areas are the areas that the robot, or the robot’s planning and navigation system, has automatically marked as not preferred in terms of navigation. They are usually found around obstacles to avoid the risk of collision.

### 3.3. AMR and KNX Interoperability

**Figure 5:**
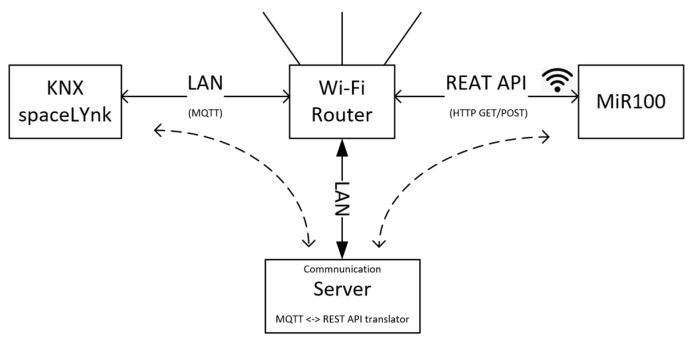
Block diagram of AMR and KNX interoperability.

By default, the AMR is connected to a local network or technology via Wi-Fi. MiR100 includes a built-in REST API based on HTTP GET/POST protocol. Using this API, it is possible to communicate with the robot, read its operational data, and assign tasks, called missions [54]. The connection of the MiR100 robot to the KNX system is made using a communication server that mediates the communication between KNX and the MiR100 robot. The communication server is connected to a wireless router to which the MiR100 mobile robot is simultaneously connected via a WIFI wireless network. Through this network, they communicate with each other using the HTTP GET/POST protocol [54]. The KNX technology is connected to the communication server via LAN using a SpaceLYnk logic controller. SpaceLYnk is used to provide visualization of operational and technical functions in buildings. At the same time, SpaceLYnk can be used as an interface between KNX technology, BACnet, DALI, Modbus, or IoT platforms (through MQTT). SpaceLynk allows providing connectivity for different types of technologies with the following technical options: IP LAN connection 10/100 Mbit, USB 2.0 (for GMS modem, EnOcean…) 5 V, 500 mA max., RS-232, Modbus (RS-485), Wi-Fi using IP connection and wireless router, KNX/EIB TP Bus. The MQTT protocol is used to ensure communication between AMR and KNX technology. Thus, the communication server (Figure 5) serves as a request translator between the mobile robot’s REST API and the MQTT protocol (supported by the SpaceLYnk module). The communication server can be implemented as a service running on a local machine or server. In certain cases, it is possible to use the services of a router or NAS system that can also serve as the local server. However, the most suitable solution seems to be the use of an embedded computer located in a rack together with a SpaceLYnk module running the translation service of the individual communication interfaces. A block diagram of the communication system is shown in Figure 5.

## 4. Living Laboratory—Smart Home Care

**Figure 6:**
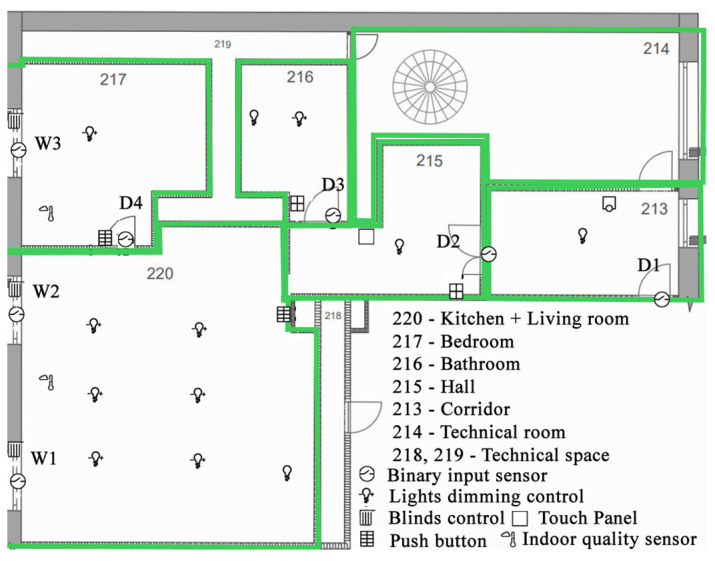
Floor plan with positioned sensors and bus buttons for controlling operational and technical functions in Smart Home Care (using the eConfigure software tool, version 1.7.5.7).

It is a functionally defined 2 + 1 apartment, so-called Living laboratory (Smart Home Care) designed for research, simulation, and data processing from the “smart” home at the VŠB TU Ostrava (Czech Republic). Specifically, the rooms are the living room with kitchen 220, bedroom 217, bathroom and toilet 216, hall 215, entrance hall 213, and technical room 214, where the switchboards are located (Figure 6). For this functionally defined part of the building, a switchboard is installed for both the power line connection and the measurement and control (MaC) equipment using KNX technology. The KNX technology provides comfortable lighting control over the DALI bus, its control, and possibly dimming. For this purpose, separate push-button controllers with signal LEDs or combined controllers with sensing of physical variables (temperature, humidity, CO_2_, and lighting intensity) are installed in the rooms. Occupancy detectors with integrated light intensity measurements are also installed in the living rooms, which, in coordination with the weather control panel, can automatically control the blinds and light intensity to ensure the light comfort of the space—based on the position of the sun and the angle of the sun’s rays. The weather station also provides other important information such as outside temperature, wind speed, presence of rain, etc. From the presence detection, it is also possible to set the parameters of space usage (lighting blocking, heating attenuation, etc.). A touch panel is installed at the entrance to the functionally defined area for user settings or system parameterization. All sensors (sensors) and controls (actuators) are connected to a common KNX bus. Ventilation and space heating are provided by an autonomous Heating, Ventilation, and Air Condition (HVAC) unit with Modbus communication (connected via a system integrator to the building control system), as well as a variable refrigerant flow (VRF) system in combination with one outdoor and several indoor units. The entire VRF system can be controlled via BMS software (Desigo CC V5.0) using a BACnet gateway down to the level of the end distribution elements. The control concept is based on a room automation station. This integrates the control logic algorithms, KNX, and DALI bus and connects the system to the higher-level BMS system. The actuators control the lighting for the kitchen and dining table as well as the bathroom fan. The kitchen ventilation is controlled by the kitchen and bathroom fans. The shutter actuators are used for the outdoor blinds and shutters.

When selecting a control system, the important criterion of the system’s ability to handle HW and SW requirements for individual control tasks is accepted. KNX technology is capable of variable expansion of HW and SW inputs/outputs. For the possibility of changing control parameters, a control panel with an LCD display is placed in anteroom 215 (Figure 6). The KNX bus is used as the communication platform. The visualization software Wiser (Embedded Systems SIA © 2021 Schneider Electric © 2021) integrates the visualization, archiving, and control of the operational and technical functions in the SHC using the SpaceLYnk controller. The ETS 6 software tool (version 6.1.0) is used for programming the KNX technology.

## 5. Experimental Part

KNX sensors MTN6005-0001 (temperature, relative humidity, and CO_2_) and MTN630719 (lighting) were used to measure the quality of the indoor environment. The CO_2_ concentration is an excellent indicator of the quality of the air and its “breathability” indoors. Since every person naturally releases a significant amount of CO_2_ by breathing (the exhaled air of an adult contains approximately 40,000 ppm CO_2_), measuring the CO_2_ concentration provides reasonably accurate information about the number of people in an enclosed space and can be easily used to regulate ventilation intensity. The location of the sensors was chosen according to CSN EN ISO 16000-26. The area of the rooms where the measurements were performed was less than 50 m^2^, therefore it was possible to use only one measurement point at a height of 1.5 m and 1 m from the wall. This is a naturally ventilated area. It was assumed that the CO_2_ concentration in the room was the same at all points. For the measurements, a five-minute interval was chosen to record the values in the SpaceLYnk controller. The measurements were carried out over a period of one month 15 March–14 April 2022. For the security area of the SHC apartment, magnetic contacts SA203 were connected to the KNX module of binary inputs MTN 644,592 to monitor the opening and closing windows (W1, W2, and W3) and doors (D1, D2, D3, and D4) (Figure 6).

### 5.1. Part 1 KNX-AMR—Localization of the Position of the Person in the SHC

**Figure 7:**
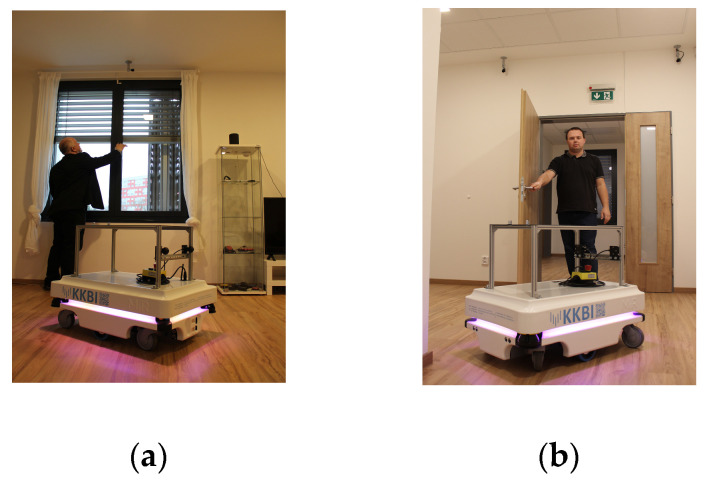
AMR locates the position of a person using an indirect method (without using cameras) based on the connection with KNX technology: (**a**) opening/closing a window (W1); (**b**) opening/closing a door (D2).

AMR locates the position of a person using an indirect method (without using cameras) based on the connection of AMR with KNX technology—opening/closing a window (W1, W2, and W3) (Figure 7a) or a door (D1, D2, D3, and D4) (Figure 7b).

### 5.2. Part 2 KNX-AMR Monitors Room Occupancy Indirectly Ahead of Time by Predicting CO_2_ Concentration Using NAR’s NN

**Figure 8:**
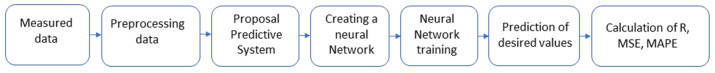
Block diagram of measured values processing with PC (processor AMD Ryzen 3 3100 4-Core Processor 3.60 GHz, Memory 16.0 GB).

The AMR indirectly monitors (without the use of a camera) the presence of a person in a room ahead of time based on the prediction of the CO_2_ concentration using the NAR NN, and then transmits the information to the AMR without the use of a camera (indirect determination of the occupancy of the monitored spaces in the SHC). The CO_2_ concentration was measured using a KNX sensor MTN6005-0001. The CO_2_ measurement range is from 300 ppm to 9999 ppm. The accuracy is for measured CO_2_ values from 300 to 1000 ppm; ±120 ppm, for measured CO_2_ values from 1000 to 2000 ppm; ±250 ppm, and for measured CO_2_ values from 2000 to 5000 ppm; ±300 ppm. Figure 8 shows a block diagram describing the processing of the measured quantities using the selected NAR and NARX NN.

The MATLAB R2020b development environment was used to create the prediction models. Specifically, the NN Time Series Tool (ntstool) in the NN Toolbox was used for this purpose. This is used exclusively for the prediction of dynamic time series, where using one or more past values of a given time series, a prediction of its future evolution is made. This tool allows a choice of three prediction models. In this paper, the NAR and NARX prediction models were used. Mean Squared Error (MSE), Mean Absolute Percentage Error (MAPE), and Spearman’s correlation coefficient R were used to evaluate the prediction (Table 2 and Table 3).

#### Nonlinear Autoregressive (NAR) Model

**Figure 9:**
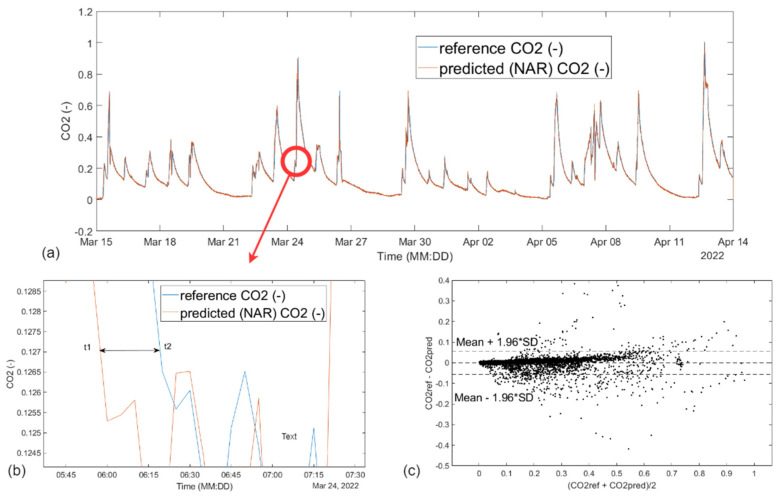
Display of (**a**) measured reference and predicted CO_2_ concentration waveform using NN NAR (10 neurons, d = 5); (**b**) larger detail of measured reference and predicted CO_2_ concentration waveform using NN NAR (10 neurons, d = 5); (**c**) Bland–Altman plot comparing predicted and reference CO_2_ waveform.

The first prediction model used was a nonlinear autoregressive NN model from MATLAB R2020b. The display of measured reference CO_2_ and predicted CO_2_ concentration waveform using NN NAR (10 neurons, d = 5) is shown in Figure 9.

It can predict future values based on past values of a given time series. Subsequently, the measured CO_2_ waveform values are divided into three sets, where 70 percent of the data is used for training the network, 15 percent for validation to better generalize the network and stop training when the generalization stops improving, and the remaining 15 percent for a test set to evaluate the network performance during and after training. The individual data are sorted into these three sets by random selection. Then, the number of neurons in the hidden layer of a given NN was chosen to be 10, and the number of output delay periods from 2 to 5.

### 5.3. Part 3 KNX-AMR Monitors Room Occupancy Indirectly Ahead of Time by Predicting CO_2_ Concentration Using a NARX NN

The AMR indirectly monitors (without the use of a camera) the presence of people in the room in advance based on the prediction of the CO_2_ concentration in advance using measured KNX technology operational variables such as indoor temperature, indoor relative humidity, indoor light intensity, window opening/closing information (W1, W2, and W3), and door opening/closing information (D1, D2, D3, and D4) using the NARX NN and then transmitting the information to the AMR.

#### Nonlinear Autoregressive with External (Exogenous) Input (NARX) Model

**Figure 10:**
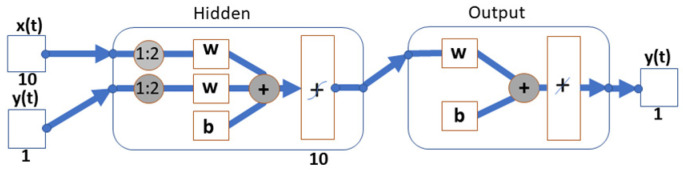
The model of the NARX network.

The second prediction model used in this work was a nonlinear autoregressive NN model with an external (exogenous) input. This can predict the future values of a time series based on its past values and other supporting data that come together at the network input. This would provide better prediction capabilities to the NN as it has more data and connections between them. Mathematically, this model can be expressed as *y*(*t*) = *f*(*x*(*t* − 1),…, *x*(*t* − *n*), *y*(*t* − 1),…, *y*(*t* − *n*)), where the CO_2_ value prediction of a time series *y*(*t*) is determined by *n* the number of values of its preceding and following time series *x*(*t*) and their *n* the number of values of their preceding (Figure 10).

**Figure 11:**
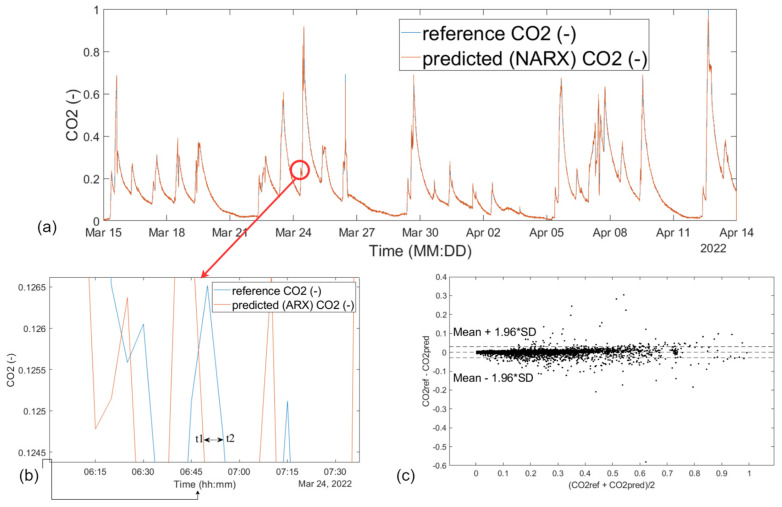
Display of (**a**) measured reference and predicted CO_2_ concentration waveform using NN NARX (10 neurons, d = 2); (**b**) larger detail of measured reference and predicted CO_2_ concentration waveform using NN NARX (10 neurons, d = 2); (**c**) Bland–Altman plot comparing predicted and reference CO_2_ waveform.

In contrast to the NAR prediction model, the NARX model has two variables, where the first contains the desired values of the CO_2_ concentration waveform and the second contains the measured values within the KNX operational measurements of indoor temperature, indoor relative humidity, indoor lighting intensity, window opening/closing information (W1, W2, and W3), and door opening/closing information (D1, D2, D3, and D4)—input external variables that represent the exogenous input of the NARX network. Other network settings are the same as in the NAR model described above. The data are also divided into three sets with a ratio of 70 percent of the data for the training set, 15 percent for the validation set, and 15 percent for the test set. The individual data are sorted into these three sets by random selection. Then, the number of neurons in the hidden layer of a given NN was selected and the number of output delay periods was set. The NARX NN model view (Figure 10) shows the number of neurons in the hidden layer, the output value delay, the activation functions at each layer, and the number of exogenous inputs entering the network. Subsequently, the NN trained using the Levenberg–Marquardt algorithm is created, and the Mean Squared Error (MSE) method is used to measure the error. After training, a table with the error results for each set is displayed. From it, it can be seen that the NARX model achieves better MSE and R results than the NAR. The second prediction NARX model used was a nonlinear autoregressive NN model from MATLAB R2020b. The display of measured reference CO_2_ and predicted CO_2_ concentration waveform using NN NAR (10 neurons, d = 5) is shown in Figure 11.

### 5.4. Part 4—Monitoring the Presence of People in the SHC Using Sensors Placed on the AMR

**Figure 12:**
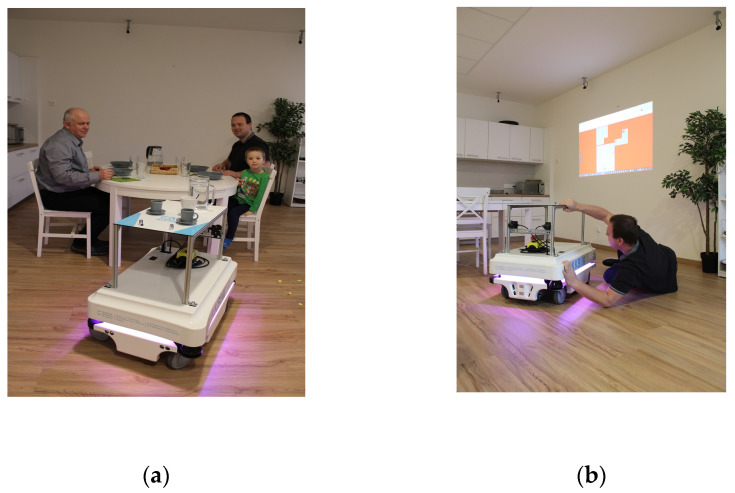
The AMR locates the position of the person using sensors placed on the AMR, specifically two lidars and ultrasonic sensors. (**a**) Mode III—be nearby: the robot is used to assist the person while eating; (**b**) Mode IV—standby: the robot is used to assist the person when trying to get up from the ground after a fall.

Monitoring the presence of people in the SHC using sensors placed on the AMR (MiR100) is shown in Figure 12. MiR100 exploits its sensor suite, mapping, and localization capabilities, along with computer vision algorithms, to recognize the presence of people in the environment it navigates. By distinguishing between free space and obstacles and employing safety protocols, the robot ensures the safety of both itself and the people in its vicinity.

The principle of people recognition relies on dynamic evaluation of changes from lidar and ultrasonic sensors, exploiting the assumption that there is no motion for fixed obstacles. In the case of detecting people or pets, AMR uses an algorithm that detects the movement of objects in space. Even if the person is not in direct motion, there is some interference with natural movement, breathing, and other biological processes.

## 6. Discussion

In this article, we present a unique proposal for the implementation of a mobile robot to KNX technology in a home environment within SHC automation to determine the presence of people and occupancy of occupied spaces in SHC using measured operational and technical variables (to determine the quality of the indoor environment) such as temperature, relative humidity, light intensity, and CO_2_ concentration and for locating occupancy in SHC spaces using magnetic contacts monitoring the opening/closing of windows and doors by indirectly monitoring occupancy without the use of cameras.

We used nonlinear autoregressive Neural Networks (NN) with NAR and NARX prediction models to predict the CO_2_ concentration waveform to transmit the information from KNX technology to mobile robots for monitoring and determining the occupancy of people in SHC with better than 98% accuracy. Enhanced predictive accuracy is possible using the optimization of algorithms [55,56,57,58,59,60,61,62] or filtration of predicted waveforms with adaptive algorithms [61,63] or with wavelet transformation [64,65]. In this article, we focused on NAR and NARX prediction models. We trained the NN NAR predicted model using the Levenberg–Marquard algorithm because it is the fastest and has standard data processing quality. For better results in training NN to predict the measured data, we could use the Bayesian Regularization algorithm, but it is slow. We did not use the Scaled Conjugate Gradient algorithm due to the poorer quality of the measured data processing. For training of the NN NARX predicted model. the properties of the algorithms described above were similar.

In our studies, in which a detailed description is provided, we dealt with the various optimization steps that could be undertaken to enhance the predictive accuracy of designed models [60,61,62,63,64]. The results of the proposed method are comparable with the state-of-the-art approaches, as summarized in Table 4.

### 6.1. Evaluation of NAR and NARX Prediction Models

#### 6.1.1. Evaluation of NAR Prediction Model

The first prediction model in this study was the NAR model. It is a very fast learning model using which a high success rate can be achieved (Table 2) in predicting the CO_2_ concentration trend (Figure 9a). The NAR prediction model allowed the optimization steps to search for the ideal setting to solve a particular problem. Despite their combinability, they are very time-consuming, since after every single setting the network has to be retrained again and evaluated whether the change produced a better result or just the opposite. One of the biggest drawbacks of this model is that the NAR model only works with historical data of a given CO_2_ concentration path and thus does not provide room for additional input data that could further improve its success rate. However, when the NAR prediction model is properly tailored to a specific CO_2_ concentration history, the model can serve as a fairly significant decision support tool regarding the presence of people in the SHC monitoring area well in advance.

#### 6.1.2. Evaluation of NARX Prediction Model

The second prediction model in this work was the NARX model. It is also one of the fast learning models and its best result in predicting the future CO_2_ concentration (Figure 11) was better than 99% (Table 3). Of course, such a high success rate can only be achieved in certain cases and with proper optimization of the prediction model. NARX can predict the future values of a time series based on its past values and other supporting data that come together at the input of the network, thus eliminating the main drawback of the NAR model. Specifically, the technical indicators should provide the network with an even better prediction success rate because of the availability of more data and the relationships between them. This was reflected in its tested model series, where it provided more consistent results. Similar to NAR, there is room for optimization in the NARX model. However, the variety of settings and the number of optimization steps, in this case, increases as it is additionally possible to optimize and adjust the calculations of technical indicators. After the analyses performed on both tested prediction models, the NARX model appears to be the best model for predicting the future CO_2_ concentration path for determining the presence of people in the SHC monitoring area. This is due to its greater variability, the larger amount of input data on which it can perform technical analyses, and more consistent results in prediction compared to the NAR model. When properly tailored and optimized to a specific problem, it can provide valuable information to investors when making decisions about the occupancy of SHC-monitored space.

### 6.2. Practical Use of AMR within SHC Using KNX Technology

The article describes the modes of interaction of AMR with people in the monitored SHC space using information about the location, presence, or occupancy of the SHC space using the connection between AMR and building automation using KNX technology.

#### 6.2.1. I. Mode—Robot Invisible

**Figure 13:**
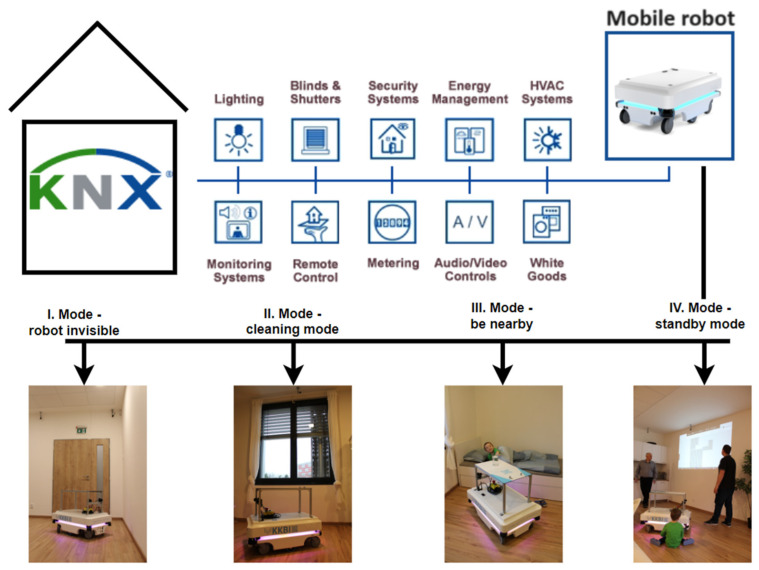
The effect of each mode.

I. Mode—robot invisible (Figure 13): the robot tries not to bother the persons in the monitored area (state—avoiding persons, parking AMR in a reserved area, and cleaning in a different room where the person is not present (vacuuming and washing the floor)); in the absence of persons, the robot receives information from KNX technology about opening/closing windows (Figure 7a), doors (Figure 7b), (CO_2_ increase/decrease), and localization of window and door contacts by AMR. Leaving/entering the apartment is an indication of the opening of the main door and decrease/increase in CO_2_ in the monitored rooms (Figure 7b).

#### 6.2.2. II. Mode—Cleaning Mode

II. Cleaning mode (Figure 13), in case of absence of persons in the apartment: binding to the door lock, lock, motion sensors, detection of opening of the main door closing, CO_2_ drops (vacuum, wash the floor, and take out the garbage)—switching on KNX using the button.

#### 6.2.3. III. Mode—Be Nearby

III. Mode—be nearby (Figure 13): the robot is used to serve people and bring food and drink (Figure 12a), (KNX button 1—kitchen-come, 2 living room-come, 3—bedroom-come, and 4 bathroom-come).

#### 6.2.4. IV. Standby Mode

IV. Standby mode (Figure 13): monitoring the presence of a person, monitoring the occupancy of the premises (localization of persons) in order to prevent possible situations (e.g., fall of a person (Figure 12b), I am not well (illness)—bring food and drink):

(a) Indirectly monitoring based on the evaluation of measured values of operational technical functions in the apartment (CO_2_ concentration, prediction of CO_2_ concentration (Figure 9a and Figure 11a) from other variables, activity (opening windows, doors, kitchen, refrigerator, starting water, starting the washing machine, dishwasher), W1, W2, W3, D1, D2, D3, and D4—localization of coordinates);
(b) Monitoring the presence of people in the SHC using sensors placed on the robot, specifically two lidars and ultrasonic sensors.

### 6.3. Robustness of the System and Measures to Mitigate Potential Failures

The mobile robot’s safety functions are managed using the utilization of Sick’s safety PLC system, known for its excellence in comparison to other systems, for example, the industrial PC. This robust safety system guarantees a comprehensive level of safety during the robot’s operation. The mobile robot is inherently capable of fully autonomous operation and is programmed to strictly adhere to safety protocols, rendering it incapable of executing any actions that contravene established safety rules.

The safety of the robot is ensured by a number of sensor systems. Essential functions such as basic navigation and spatial orientation are facilitated using a pair of lidars. Nevertheless, lidar technology has inherent limitations, including the inability to detect objects such as transparent obstacles and vulnerability to sun-induced interference. When faced with sun interference, the robot’s mobility ceases immediately until normal conditions are restored. To address the challenge of transparent obstacles, the robot is equipped with ultrasonic sensors, providing reliable obstacle detection capabilities. Additionally, proximity sensors are integrated into the robot to monitor items, such as small objects on the floor, contributing to its overall safety.

An additional layer of safety is realized using the robot’s navigation system, which relies on a predefined map. This map can encompass various zones with distinct attributes, including one-way streets, restricted areas, and non-preferred zones. The robot is programmed to refrain from entering these zones, even when physically feasible. This navigation, while complex, is seamlessly managed by the mobile robot’s planning and navigation system, which takes these defined areas into account.

Originally designed for industrial applications, the mobile robot’s safety system is intentionally engineered to facilitate its safe coexistence with human environments, emphasizing its adaptability and versatility.

## 7. Conclusions

The article presents its own unique implementation of AMR with connectivity to KNX technology in the home environment within SHC automation for determining the presence of people and occupancy of occupied spaces in SHC using measured operational and technical variables, (for determining the quality of the indoor environment) such as temperature, relative humidity, lighting intensity, and CO_2_ concentration and for locating the presence of people in SHC spaces using magnetic contacts monitoring the opening of windows via the indirect method of monitoring the presence of people and occupancy of monitored SHC spaces without the use of cameras.

In this paper, the implementation of a learned NN NARX and NAR with a learning Levenberg–Marquard algorithm for predicting the CO_2_ concentration waveform in advance to forward information from KNX AMR technology for monitoring and determining occupancy in Smart Home Care with an accuracy of better than 98%.

The proposed architecture was applied to an existing decentralized KNX technology in a Smart Home environment for the purpose of occupancy monitoring of individual SHC rooms.

In the experimental part, the localization of the person’s position by exchanging information between KNX and AMR technology was described, and an example of using NN NAR to monitor room occupancy indirectly with time advance by predicting CO_2_ concentration was presented.

In the next section, a method for determining the presence of persons using measured variables by KNX technology was described, where AMR monitors room occupancy indirectly in advance by predicting CO_2_ concentration using a NARX NN.

The experiments also described the monitoring of occupant presence in SHC using sensors placed on the AMR.

In future studies, the authors will focus on the detailed description of occupant localization and occupant presence determination using AMR in conjunction with KNX technology designed to build automation.

## Figures and Tables

**Table 1:** Studies that investigate the application of robots within Smart Home Care.

Topic of the Article	Ref. No.	Observations
Use of Social Companion Robot (SCR) for Adults with Motor Disabilities (MD).	[16]	SCR, MD
A Multirobot (MR) System in an Assisted Home Environment (AHE) to Support the Elderly in Their Daily Lives (DL).	[17]	MR, Support elderly, AHE, DL
Personalized home-care (PHC) robot support for the elderly.	[18]	R, PHC
Artificial Intelligence-Based Smart Comrade Robot for Elders Healthcare (HC) with Strait Rescue System.	[19]	R, AI
Healthcare Live-in Prognostic Robot (or HLPR).	[20]	R, HC, HLPR
Automatic pathological gait recognition (PGR) by a mobile robot using ultrawideband-based localization and a depth camera.	[21]	R, PGR
Bridging gaps in the design and implementation of socially assistive technologies (SAT) for dementia care: the role of occupational therapy.	[22]	R, Dementia, SAT
Discrete HMM for Visualizing Domiciliary Human Activity (HA) Perception and Comprehension.	[23]	R, HMM, HA
The AI devices utilized in elderly healthcare were summarized as robots.	[24]	R, AI, HC
Virtual reality technologies (VRT), smart wearables, and robots were used to provide telerehabilitation services (TRS)	[25]	R, VRT, R, TRS
Multi-Agent Interaction (MAI) to Assist Visually Impaired (AVI) and Elderly People	[26]	R, MAI, AVI
Analysis of IoT Cloud Security Computerization Technology Based on Artificial Intelligence Robot, SH	[27]	R, AI, IoT, SH
A microservice architecture solution for rapid prototyping (RP) of robotic solutions to COVID-19 challenges in care facilities	[28]	R, RP
Evaluation and intention to use the interactive robotic kitchen system AuRorA in older adults—food preparation	[29]	R, food preparation
Home Based Monitoring for Smart Health-Care (SHC) Systems: A Survey	[30]	R, SHC
A Smart Home (SH) Based on Multi-heterogeneous Robots and Sensor Networks for Elderly Care (EC)	[31]	R, SH, EC
A Task Allocation Approach of Multi-Heterogeneous Robot System for Elderly Car (EC)	[32]	R, EC

**Table 2:** Calculated values of MSE, R, and MAPE NAR for the number of hidden neurons 10.

Number of Delays d	MSE	R [%]	MAPE
d2	4.439 × 10^−5^	99.892	0.0458
d3	1.061 × 10^−5^	99.753	0.0624
d4	4.342 × 10^−5^	99.895	0.0803
d5	2.923 × 10^−5^	99.918	0.0847
d6	4.821 × 10^−5^	99.882	0.0904

**Table 3:** Calculated MSE, R, and MAPE values of NN NARX for the number of hidden neurons 10, number of delays d = 2, and d = 4.

**Number of Hidden Neurons 10** **Number of Delays d = 2**	**MSE**	**R [%]**	**MAPE**
input2NARX Temp, rH	8.737 × 10^−5^	99.782	0.0514
input3NARX including Temp, rH, E	3.746 × 10^−5^	99.905	0.0745
input4NARX including Temp, rH, E, D1–D4	3.442 × 10^−5^	99.911	0.0544
input5NARX including Temp, rH, E, D1–D4, W1–W3	3.322 × 10^−5^	99.913	0.0565
**Number of Hidden Neurons 10** **Number of Delays d = 4**	**MSE**	**R [%]**	**MAPE**
input2NARX Temp, rH	1.797 × 10^−4^	99.523	0.0788
input3NARX including Temp, rH, E	2.104 × 10^−5^	99.466	0.0756
input4NARX including Temp, rH, E, D1–D4	2.984 × 10^−5^	99.922	0.0821
input5NARX including Temp, rH, E, D1–D4, W1–W3	4.568 × 10^−5^	99.900	0.1011

**Table 4:** Comparison with state-of-the-art approaches for occupancy estimation and CO_2_ sensing.

Topic of the Article	Observations	Accuracy (%)
Accurate occupancy detection of an office room from light, temperature, humidity and CO_2_ measurements using statistical learning models [55].	Statistical models	95–99%
Data Collection Period and Sensor Selection Method for Smart Building Occupancy Prediction [56]	machine learning classifier algorithms	90%
Opportunistic occupancy-count estimation using sensor fusion [57]	Wi-Fi access points, CO_2_ sensors, PIR motion detectors, and plug and light electricity load meters	83%
Office Building’s Occupancy Prediction Using Extreme Learning Machine Model with Different Optimization Algorithms [58]	Machine learning	-
A vision-based deep learning approach for the detection and prediction of occupancy heat emissions for demand-driven control solutions [59]	Deep Learning Influenced Profile	>80%
Occupancy Detection in Smart Home Space Using Interoperable Building Automation Technologies [60].	ANN Levenberg–Marquardt algorithm, Bayesian regularization algorithm, Scaled conjugate gradient algorithm	>84%
Design of a New Method for Detection of Occupancy in the Smart Home Using an FBG Sensor [61].	ANN SCG	>90%
Occupancy prediction through Markov based feedback recurrent neural network (M-FRNN) algorithm with WiFi probe technology [62]	M-FRNN	>80%
Novel Proposal for Prediction of CO_2_ Course and Occupancy Recognition in Intelligent Buildings [63].	Linear Regression, Neural Networks, and Random Tree Adaptive filtration	>90%
Wavelet-Based Filtration Procedure for De-noising the Predicted CO_2_ Waveforms [64]	wavelet transformation	>98%
Using the IBM SPSS SW Tool with Wavelet Transformation for CO_2_ Prediction [65].	Radial Basis Function (RBF) method	>95%

## Data Availability

The data presented in this study are available on request from the corresponding author. The data are not publicly available due to privacy.

## References

[1] Anjum T., Lawrence S., Shabani A. Augmented Reality and Affective Computing on the Edge Makes Social Robots Better Companions for Older Adults. Proceedings of the 2nd International Conference on Robotics, Computer Vision and Intelligent Systems (ROBOVIS).

[2] Abdi S., de Witte L., Hawley M. (2020). Emerging Technologies With Potential Care and Support Applications for Older People: Review of Gray Literature. JMIR Aging.

[3] Abd Malik S., Aburahmah L., Azuddin M. An Exploratory Study on the Use of Social Companion Robot for Adults with Motor Disabilities. Proceedings of the 6th International Conference of Reliable Information and Communication Technology (IRICT).

[4] Abrar M.M., Islam R., Shanto M.A.H. An Autonomous Delivery Robot to Prevent the Spread of Coronavirus in Product Delivery System. Proceedings of the 11th IEEE Annual Ubiquitous Computing, Electronics and Mobile Communication Conference (UEMCON).

[5] Arias-de-Reyna E., Closas P., Dardari D., Djuric P.M. (2018). Crowd-Based Learning of Spatial Fields for the Internet of Things From harvesting of data to inference. IEEE Signal Process. Mag..

[6] Bai T., Fan Z., Liu M.Q., Zhang S.L., Zheng R.H. Multiple Waypoints Path Planning for a Home Mobile Robot. Proceedings of the 9th International Conference on Intelligent Control and Information Processing (ICICIP).

[7] Barber R., Ortiz F.J., Garrido S., Calatrava-Nicolas F.M., Mora A., Prados A., Vera-Repullo J.A., Roca-Gonzalez J., Mendez I., Mozos O.M. (2022). A Multirobot System in an Assisted Home Environment to Support the Elderly in Their Daily Lives. Sensors.

[8] Bardaro G., Daga E., Carvalho J., Chiatti A., Motta E. (2022). Introducing a Smart City Component in a Robotic Competition: A Field Report. Front. Robot. AI.

[9] Bat-Erdene B.O., Saver J.L. (2021). Automatic Acute Stroke Symptom Detection and Emergency Medical Systems Alerting by Mobile Health Technologies: A Review. J. Stroke Cerebrovasc. Dis..

[10] Berrezueta-Guzman J., Pau I., Martin-Ruiz M.L., Maximo-Bocanegra N. (2021). Assessment of a Robotic Assistant for Supporting Homework Activities of Children With ADHD. IEEE Access.

[11] Bodur G., Gumus S., Gursoy N.G. (2019). Perceptions of Turkish health professional students toward the effects of the interne of things (IOT) technology in the future. Nurse Educ. Today.

[12] Brunete A., Gambao E., Hernando M., Cedazo R. (2021). Smart Assistive Architecture for the Integration of IoT Devices, Robotic Systems, and Multimodal Interfaces in Healthcare Environments. Sensors.

[13] Cao W.J., Yu H.L., Wu X.Y., Li S.J., Meng Q.L., Chen C.J. (2021). Development and Evaluation of a Rehabilitation Wheelchair with Multiposture Transformation and Smart Control. Complexity.

[14] Cavallo F., Limosani R., Fiorini L., Esposito R., Furferi R., Governi L., Carfagni M. (2018). Design impact of acceptability and dependability in assisted living robotic applications. Int. J. Interact. Des. Manuf. Ijidem.

[15] Information Technology—Home Electronic System (HES) Architecture. https://www.iso.org/obp/ui/en/#iso:std:iso-iec:14543:-3-4:ed-1:v1:en.

[16] Calatrava-Nicolas F.M., Gutierrez-Maestro E., Bautista-Salinas D., Ortiz F.J., Gonzalez J.R., Vera-Repullo J.A., Jimenez-Buendia M., Mendez I., Ruiz-Esteban C., Mozos O.M. (2021). Robotic-Based Well-Being Monitoring and Coaching System for the Elderly in Their Daily Activities. Sensors.

[17] Gochoo M., Alnajjar F., Tan T.H., Khalid S. (2021). Towards Privacy-Preserved Aging in Place: A Systematic Review. Sensors.

[18] Di Napoli C., Ercolano G., Rossi S. (2022). Personalized home-care support for the elderly: A field experience with a social robot at home. User Model. User-Adapt. Interact..

[19] Dilip G., Guttula R., Rajeyyagari S., Hemalatha S., Pandey R.R., Bora A., Kshirsagar P.R., Khanapurkar M.M., Sundramurthy V.P. (2022). Artificial Intelligence-Based Smart Comrade Robot for Elders Healthcare with Strait Rescue System. J. Healthc. Eng..

[20] Iantorno M., Intson C. Lifecycle: A Speculative Fiction on Healthcare Automation. Proceedings of the 17th Annual ACM/IEEE International Conference on Human-Robot Interaction (HRI).

[21] Jun K., Oh S., Lee S., Lee D.W., Kim M.S. Automatic pathological gait recognition by a mobile robot using ultrawideband-based localization and a depth camera. Proceedings of the 31st IEEE International Conference on Robot and Human Interactive Communication (RO-MAN)—Social, Asocial, and Antisocial Robots.

[22] Koh W.Q., Heins P., Flynn A., Asl A.M., Garcia L., Malinowsky C., Brorsson A. (2022). Bridging gaps in the design and implementation of socially assistive technologies for dementia care: The role of occupational therapy. Disabil. Rehabil. Assist. Technol..

[23] Kuan T.W., Tseng S.P., Chen C.W., Wang J.F., Sun C.A. (2022). Discrete HMM for Visualizing Domiciliary Human Activity Perception and Comprehension. Appl. Sci..

[24] Ma B.X., Yang J., Wong F.K.Y., Wong A.K.C., Ma T.T., Meng J.A., Zhao Y., Wang Y.G., Lu Q. (2023). Artificial intelligence in elderly healthcare: A scoping review. Ageing Res. Rev..

[25] Moulaei K., Sheikhtaheri A., Nezhad M.S., Haghdoost A., Gheysari M., Bahaadinbeigy K. (2022). Telerehabilitation for upper limb disabilities: A scoping review on functions, outcomes, and evaluation methods. Arch. Public Health.

[26] Oliveira J.D., Engelmann D.C., Kniest D., Vieira R., Bordini R.H. (2022). Multi-Agent Interaction to Assist Visually-Impaired and Elderly People. Int. J. Environ. Res. Public Health.

[27] Padmaavathy P.A., Bharathi S.S., Kumar K.A., Prasad C.V.S., Ramachandran G. Analysis of IoT Cloud Security Computerization Technology Based on Artificial Intelligence. Proceedings of the 3rd International Conference on Image Processing and Capsule Networks (ICIPCN).

[28] Panchea A.M., Letourneau D., Briere S., Hamel M., Maheux M.A., Godin C., Tousignant M., Labbe M., Ferland F., Grondin F. (2022). OpenTera: A microservice architecture solution for rapid prototyping of robotic solutions to COVID-19 challenges in care facilities. Health Technol..

[29] Perotti L., Strutz N. (2022). Evaluation and intention to use the interactive robotic kitchen system AuRorA in older adults. Zeitschrift Fur Gerontologie Und Geriatrie.

[30] Shilvya J.A., George S.T., Subathra M.S.P., Manimegalai P., Mohammed M.A., Jaber M.M., Kazemzadeh A., Al-Andoli M.N. (2022). Home Based Monitoring for Smart Health-Care Systems: A Survey. Wirel. Commun. Mob. Comput..

[31] Zhang T.Q., Zhao D.H., Yang J.Y., Wang S.Y., Liu H.D. (2022). A Smart Home Based on Multi-heterogeneous Robots and Sensor Networks for Elderly Care. Intell. Robot. Appl. (Icira 2022) Pt I.

[32] Zhao D.H., Yang C.H., Zhang T.Q., Yang J.Y., Hiroshi Y. (2022). A Task Allocation Approach of Multi-Heterogeneous Robot System for Elderly Care. Machines.

[33] Lopez-Aguilar A.A., Navarro-Tuch S.A., Bustamante-Bello M.R., Izquierdo-Reyes J., Curiel-Ramirez L.A. Interpretation and Emulation for Telegrams of the KNX Standard on MATLAB Simulink. Proceedings of the IEEE International Conference on Mechatronics, Electronics and Automotive Engineering (ICMEAE).

[34] Seifried S., Gridling G., Kastner W. KNX IPv6: Design Issues and Proposed Architecture. Proceedings of the 2017 IEEE 13th International Workshop on Factory Communication Systems (Wfcs 2017).

[35] Toylan M.Y., Cetin E. (2019). Design and application of a KNX-based home automation simulator for smart home system education. Comput. Appl. Eng. Educ..

[36] Wang X.J., Wang Y. (2013). Research and Implementation of Data Link Layer in KNX Communication Protocol Stack. Proceedings of the 2013 Chinese Intelligent Automation Conference: Intelligent Automation & Intelligent Technology and Systems.

[37] Sowa S. Increasing the Energy Efficiency of Hybrid RES Installations Using KNX System. Proceedings of the 4th International Conference on Renewable Energy Sources (ICORES).

[38] Ajao L.A., Agajo J., Umar B.U., Agboade T.T., Adegboye M.A. Modeling and Implementation of Smart Home and Self-control Window using FPGA and Petri Net. Proceedings of the 2020 IEEE Pes Ias Powerafrica Conference.

[39] Liu P.F., Zhang W.F., Wu C.X., Sun X.M. (2021). Home automation design based on STM32. Chin. J. Liq. Cryst. Disp..

[40] Liu Y. (2021). Simulation of art design of indoor furnishings based on FPGA and internet of things system. Microprocess. Microsyst..

[41] Malik S., Lee K., Kim D. (2020). Optimal Control Based on Scheduling for Comfortable Smart Home Environment. IEEE Access.

[42] Mandaric K., Skocir P., Jezic G. Context-based System for User-Centric Smart Environment. Proceedings of the 28th International Conference on Software, Telecommunications and Computer Networks (SoftCOM).

[43] Yao K.C., Huang W.T., Wu C.C., Chen T.Y. (2021). Establishing an AI Model on Data Sensing and Prediction for Smart Home Environment Control Based on LabVIEW. Math. Probl. Eng..

[44] Zou Z.Y., Wang Y., Wang L., Wu X.W., Xu C., Zhou M. Design of smart home controller based on raspberry PI. Proceedings of the 2020 IEEE 5th Information Technology and Mechatronics Engineering Conference (Itoec 2020).

[45] Hao H.B., Dai F.Z., Wen H.K., Zhao J.C. Research on the Smart Home Design based on Single-chip Microcomputer. Proceedings of the 2020 International Conference on Artificial Life and Robotics (Icarob2020).

[46] Chang C.Y., Guo S.J., Hung S.S., Lin Y.T. (2019). Performance Analysis of Indoor Smart Environmental Control Factors: Using Temperature to Control the Rate of Formaldehyde Emission. IEEE Access.

[47] De Schepper T., Vanhulle A., Latre S. Dynamic BLE-based fingerprinting for location-aware smart homes. Proceedings of the 2017 IEEE Symposium on Communications and Vehicular Technology (SCVT).

[48] Hsu H.P., Yu K.M., Ouyang W., Xu C.J. Constructing a Smart Home Control System with the Internet of Things. Proceedings of the 7th IEEE Asia-Pacific Conference on Antennas and Propagation (APCAP).

[49] Rinaldi A., Roccotelli M., Fanti M.P. A Decision Support System for Comfort Optimization in a Smart Retirement Home. Proceedings of the IEEE International Conference on Systems, Man and Cybernetics (SMC).

[50] Hong P.J., Liu H., Yan Z.G., Qian Z.Q., Wu K., Bi Z.M. Research of Home Environment Surveillance System Based on Wireless Sensor Network. Proceedings of the 2017 17th IEEE International Conference on Communication Technology (Icct 2017).

[51] Peng J.S., Ye H.M., He Q.W., Qin Y., Wan Z.W., Lu J.X. (2021). Design of Smart Home Service Robot Based on ROS. Mob. Inf. Syst..

[52] Yang T., Zhao L.Y., Li W., Wu J.Z., Zomaya A.Y. (2021). Towards healthy and cost-effective indoor environment management in smart homes: A deep reinforcement learning approach. Appl. Energy.

[53] (2021). User Guide for MiR100 Autonomous Mobile Robots. https://gibas.nl/wp-content/uploads/2021/01/mir100-user-guide_31_en.pdf.

[54] (2022). MIR100 REST API. https://www.jugard-kuenstner.de/fileadmin/daten/Downloads/Intralogistik/MiR_Transportsystem/MiR100_MiR200/MiR_Rest-API.pdf.

[55] Candanedo L.M., Feldheim V. (2016). Accurate occupancy detection of an office room from light, temperature, humidity and CO_2_ measurements using statistical learning models. Energy Build..

[56] Haidar N., Tamani N., Nienaber F., Wesseling M.T., Bouju A., Ghamri-Doudane Y. Data Collection Period and Sensor Selection Method for Smart Building Occupancy Prediction. Proceedings of the 89th IEEE Vehicular Technology Conference (VTC Spring).

[57] Hobson B.W., Lowcay D., Gunay H.B., Ashouri A., Newsham G.R. (2019). Opportunistic occupancy-count estimation using sensor fusion: A case study. Build. Environ..

[58] Motuziene V., Bielskus J., Lapinskiene V., Rynkun G. (2021). Office Building’s Occupancy Prediction Using Extreme Learning Machine Model with Different Optimization Algorithms. Environ. Clim. Technol..

[59] Tien P., Wei S.Y., Calautit J.K., Darkwa J., Wood C. (2020). A vision-based deep learning approach for the detection and prediction of occupancy heat emissions for demand-driven control solutions. Energy Build..

[60] Vanus J., Martinek R., Danys L., Nedoma J., Bilik P. (2022). Occupancy Detection in Smart Home Space Using Interoperable Building Automation Technologies. Hum. Centric Comput. Inf. Sci..

[61] Vanus J., Nedoma J., Fajkus M., Martinek R. (2020). Design of a New Method for Detection of Occupancy in the Smart Home Using an FBG Sensor. Sensors.

[62] Wang W., Chen J.Y., Hong T.Z., Zhu N. (2018). Occupancy prediction through Markov based feedback recurrent neural network (M-FRNN) algorithm with WiFi probe technology. Build. Environ..

[63] Vanus J., Gorjani O.M., Bilik P. (2019). Novel Proposal for Prediction of CO_2_ Course and Occupancy Recognition in Intelligent Buildings within IoT. Energies.

[64] Vanus J., Fiedorova K., Kubicek J., Gorjani O.M., Augustynek M. (2020). Wavelet-Based Filtration Procedure for Denoising the Predicted CO_2_ Waveforms in Smart Home within the Internet of Things. Sensors.

[65] Vanus J., Kubicek J., Gorjani O.M., Koziorek J. (2019). Using the IBM SPSS SW Tool with Wavelet Transformation for CO_2_ Prediction within IoT in Smart Home Care. Sensors.

